# How the monarch got its spots: Long-distance migration selects for larger white spots on monarch butterfly wings

**DOI:** 10.1371/journal.pone.0286921

**Published:** 2023-06-21

**Authors:** Andrew K. Davis, Brenden Herkenhoff, Christina Vu, Paola A. Barriga, Mostafa Hassanalian

**Affiliations:** 1 Odum School of Ecology, University of Georgia, Athens, GA, United States of America; 2 Department of Mechanical Engineering, New Mexico Tech, Socorro, NM, United States of America; 3 Department of Plant Biology, University of Georgia, Athens, GA, United States of America; Southeastern Louisiana University, UNITED STATES

## Abstract

Elucidating the adaptations that promote flight in animals can aid the understanding of evolution and species divergence, and/or provide inspiration for aerospace engineering and the design of better aerial vehicles. The famed long-distance migration of monarch butterflies in North America still holds many questions and opportunities for inspiration. For example, there is little research on whether the monarch’s primary wing colors themselves (black, orange, or white) have any aerodynamic or migration function. Dark colors on wings of other animals have recently been shown to aid flight by enhancing solar absorption, which reduces drag forces. However, too much black surface could be problematic for monarchs, which are exposed to increasing amounts of solar energy along their flightpath. This paper describes the results of two related investigations that attempt to elucidate the importance of wing color to the monarch migration. By measuring the color proportions of nearly 400 monarch wings collected at different stages of their journey, we found, surprisingly, that successful migrants tended to have less black on their wings (about 3% less), but also more white pigment (about 3% more); monarchs have a band of light-colored marginal wing spots. Second, image analysis of museum specimens revealed migratory monarchs had significantly larger white spots, proportional to the wing area, than most non-migratory, New World Danaid butterflies, which argues spot size has evolved along with migratory behavior. Combined, these findings strongly suggest that the long-distance migration itself selects for larger white spots every fall, so that only those individuals with large spots will survive to pass on their genes. Further experimental work is needed to elucidate how the spots aid the migration, but it is possible that they enhance aerodynamic efficiency; other work by the authors demonstrates how alternating white and black pigment on wings can reduce drag. These results will serve as a useful starting point for such endeavors, which should improve understanding of one of the world’s most fascinating animal migrations, and also provide practical knowledge for the field of aerospace engineering.

## Introduction

**Fig 1:**
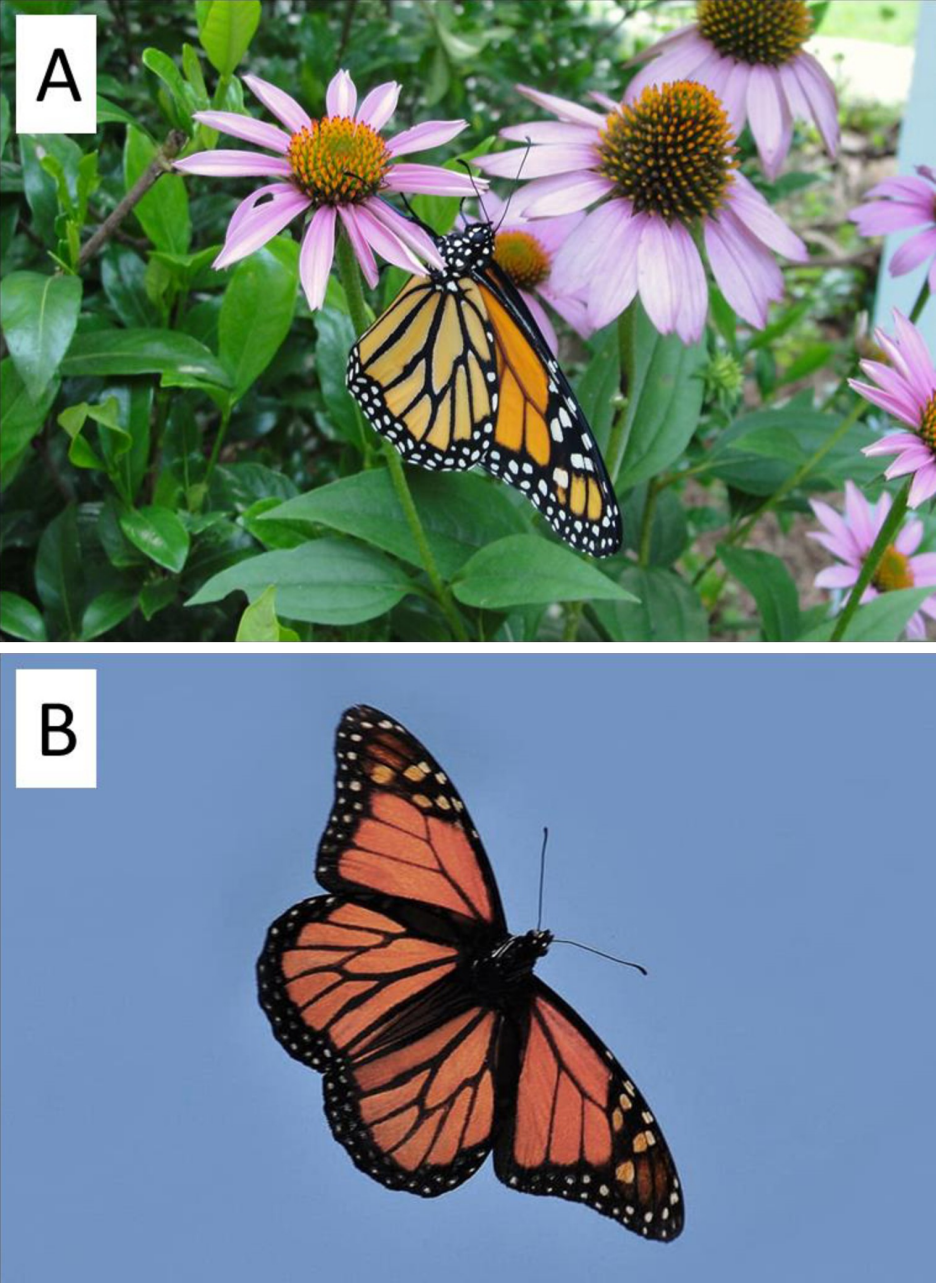
A) Monarch butterfly, *Danaus plexippus*, nectaring, in Nova Scotia, Canada on July 11, 2015. Photo by Pat Davis. B) Photo of a monarch in soaring flight, taken by John Blair. Note the position of the forewings, which are swept back during soaring.

Animals capable of flight have been shaped, sometimes quite literally, by the forces of natural selection acting on their flight structures. For terrestrial animals with long-distance migrations, like migrating birds, bats and insects, these selective forces are especially strong, because the cost of not succeeding in their migration is often death [1–4]. Thus, flying migrants are uniquely adapted for undertaking long-distance flights, with many of these adaptations being investments in highly efficient and aerodynamic wing designs [5–7]. Knowledge of these adaptations can help to inspire improvements to human-made aerial machines. In the insect world, such knowledge could be gained by taking a close look at the monarch butterfly, *Danaus plexippus* (Fig 1). Monarchs in North America, which weigh less than a paper clip, undertake one of the longest migrations of any insect in the world, travelling as much as 4000km each fall to get to their winter destinations [8, 9]. The vast majority of the North American population attempts to reach selected mountaintop sites in central Mexico, while a small fraction west of the Rocky Mountains takes a shorter route to winter in trees along the California coast. The journey of the eastern cohort takes at least two months, and this migration is fraught with uncertainty and risk, including natural and anthropogenic impacts [10–13], such that only the hardiest monarchs are successful. This migration has long fascinated scientists, even before the wintering location was known [14–16], especially given the fact that the journey is completed by individuals who have never been to the destination.

Since the Mexican overwintering sites were discovered in 1975 there has been considerable (entomological) research into understanding how such a small animal can fly so far. Some of this work has involved examining the physiological adaptations of monarchs [17–22], including their neural function and capacity, which allows them to navigate across a continent using multiple cues [23, 24]. Other work has focused on their physical adaptations, such as their wing morphology [7, 25–28]. Some research has even found that the shade (hue) of their orange wing pigment is correlated with migration success, and/or flight ability [29, 30]. This finding, that variation in pigmentation hue predicts migration success, appears to be unique within the Lepidoptera. In these cases, it is likely that the darkness of their orange color serves as a good indicator of their ability to produce pigment [31, 32], which is correlated with other fitness traits, such as flying ability.

Despite all the research into the factors that contribute to the migration success of monarchs, and even the aforementioned studies of pigmentation, few have questioned if the primary wing colors themselves (black, orange or white) have any aerodynamic function, or otherwise have an impact on the migration success [but see 29]. Meanwhile, a growing body of (aerospace engineering) literature is showing how certain wing colors can influence aerial performance of other flying animals [33, 34], essentially by affecting the amount of air resistance or drag. Specifically, a darkly-colored wing surface more readily absorbs solar energy, and this heats up the boundary layer of air immediately above it (more so than a light-colored wing), thereby creating a pocket of warm air which the wing can pass through more readily (warm air has less resistance). As such, dark colors on wings can substantially improve aerodynamic efficiency of flying animals because of their reduced drag [33, 35]. This finding appears to be a widespread phenomenon, as even dark colors on aquatic animals reduce drag during swimming, by the same principle [36]. This phenomenon has tremendous implications both for understanding animal flight, but also for improving aerospace technology. It is possible that monarch butterflies make use of this phenomenon; preliminary work examining a small number of monarch specimens, combined with computer simulations, strongly indicated that the darkly-pigmented regions of monarch wings have this same aerodynamic function [37]. In addition, one study of monarch spring migration appeared to show how more melanized monarchs travel farther [29], which also suggests melanization has a positive influence on flight ability. The current study was initiated to gather additional evidence pertaining to this phenomenon.

Monarchs, and their New World Danaid relatives, all have a similar basic color pattern on their wings (Fig 1), with an orange base, or background, and a thick black marginal stripe along the trailing edge. The position of this black band around the edge could be indicative of a role in aerial performance, in a manner consistent with the examples above (heating the air around the wing edge to provide less resistance). Also, a series of white, or sometimes pale orange spots are also arranged throughout the black margins (Fig 1). The function of these spots is not known, but their non-random, uniform arrangement specifically within the black margins suggests a purpose. It is possible that these spots have a thermally-related function, since, in the animal kingdom, white integument (fur, scales, hair) often is associated with a need to avoid excessive solar absorption and heat gain [38–40]. In butterflies in particular, a prior study of *Anartia fatima*, a species with prominent white stripes on its wings, demonstrated how the light-colored stripes help to reduce heat absorption and/or overheating [41]. By extension then, if white pigment in butterflies serves to reduce solar heat gain, the position of the white spots on monarch wings (embedded within the outer black region) may serve to counteract the thermal energy absorption of the black color. Alternatively, the fact that the white spots are regularly-spaced within the black margin (leading to alternating white and black pigment zones) could be a way to create alternating zones of heating and cooling (when exposed to solar energy), which could create micro-eddies of air currents above the wing.

**Fig 2:**
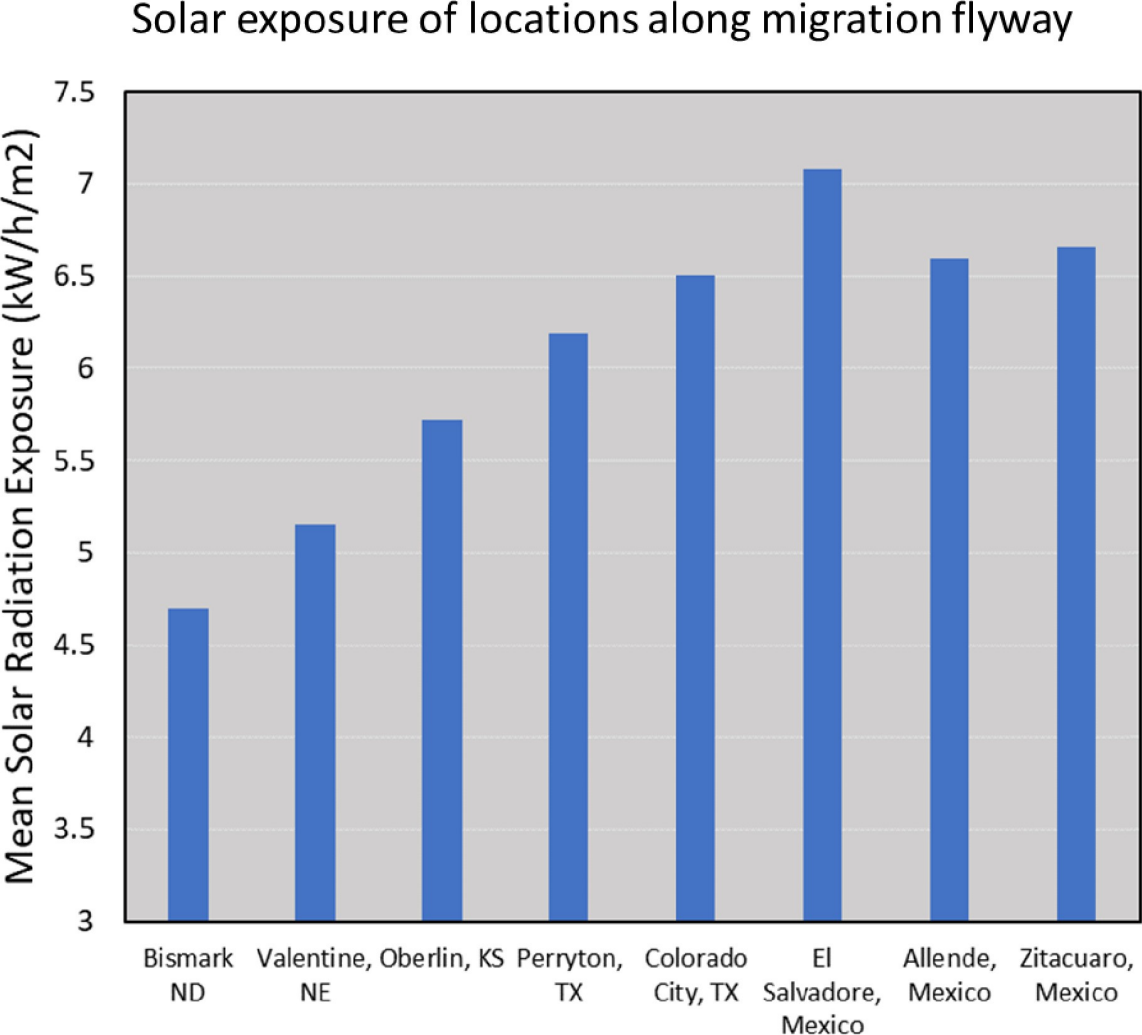
Visualization of solar exposure levels experienced by fall-migrating monarchs in eastern North America as they travel to the winter destination in central Mexico. Selected locations along the flyway were chosen for this graph, and their average solar radiation at ground level was determined from the National Solar Radiation Database (https://nsrdb.nrel.gov/).

Given the above considerations, and our prior work showing the impact of wing color on animal flight [33, 35], we reasoned that monarchs would likewise have traits that take advantage of solar exposure, for improving aerodynamic performance. In fact, consider that migrating monarchs are exposed to high levels of solar exposure during their fall migrations, especially given that a large proportion of their time in the air is spent soaring (with wings spread, as in Fig 1B), and at high altitudes [42–44]. Moreover, the intensity of solar exposure actually increases during their journey, since the flyway naturally traverses continental regions of low exposure to higher exposure (Fig 2). Also consider that adult monarchs can vary in the amount of wing surface covered by black pigment [45–48], and, that females tend to have greater amounts of black than males [49]. Therefore, if pigmentation does impact migration performance (positively or negatively), this knowledge could prove useful in interpreting sex-based differences in migration success [50], or even regional differences in successful migrations [51].

This paper describes the results of two related investigations that address the question of whether any of the monarch wing colors (black, orange, or white) have importance to the long-distance migration. We had few expectations going into this project, partially from a lack of comparable literature, but also because of the opposing lines of thought listed above relating to the black pigment. On the one hand, if black surfaces provide an aerodynamic advantage, then one could expect successful migrants to have greater amounts of black [29]. On the other hand, the high levels of solar exposure experienced during the journey may mean that heavily-melanized individuals would fare poorly. Regarding the orange pigment, aside from the aforementioned research on how the *shade* of orange is important for monarch fitness [29, 30], there is no prior information on how having more or less surface coverage of orange would be of any advantage during flight. Finally, we know that white pigment has a clear role in reducing solar heat gain in butterflies and animals, though going into this project, we were not aware of how this role would be relevant to the migration.

The first stage of our project was a retrospective examination of wing images (computer scans of specimens) from wild monarchs that had been collected at three points along the southward fall migration flyway, where we asked if the relative coverage of any of the three primary wing colors (orange, black or white) changes along the journey, which would signify selection for certain color patterns that are advantageous for migration. Based on the results of that exercise, which showed, surprisingly, that the amount of white wing pigment is somehow important, we next examined a collection of images of preserved museum specimens of monarchs and their non-migratory relatives of the New World, to ask if the size of white wing spots is associated with a migratory lifestyle across species. Collectively, these investigations provide novel insights into the ongoing efforts to uncover the secrets of the monarch migration and shed light into the role of colors for aerodynamic performance of flying species. In addition, these findings may prove useful in the growing engineering field of “bioinspiration,” by demonstrating how basic color patterns could enhance long-distance flying machine performance.

## Materials and methods

### Butterfly specimens

This project involved measurements of monarchs and related Danaid butterflies using image analysis techniques, and using collections of preserved specimens which had been scanned or photographed as part of other projects by the lead author, or from prior collaborations with colleagues. First, we utilized images of nearly 400 wild monarchs that had been collected during the summer breeding period, the fall migration, or at the Mexican overwintering sites by the lead author or colleagues. Sample sizes, year of collection, and regions are shown in Table 1. The nature of the projects is not important here, since all of these monarchs were collected as adults in the wild, had been similarly scanned after collection to produce a digital copy of their wings (using a Hewlett Packard 2300c Scanjet flatbed scanner [Hewlett Packard, Palo Alto, CA]), and with no image manipulation or color correction. What is important is that these monarchs were collected during three distinct life stages (i.e. breeding, migration or overwintering). Our intent here was to evaluate how certain wing pigmentation patterns (below) vary across the three life stages, which would point to their importance for migration success within monarchs.

**Table 1:** Summary of sample sizes for measurements of monarch wing pigmentation. All monarchs had been collected as part of other projects and their wings had been scanned by one of the authors (Davis) to create a digital image for measurement.

Stage/Location	Year	# specimens
Summer Breeding—Minnesota	1996	39
Summer Breeding—South Dakota	2012	33
Fall Migrating—Georgia	2005	44
Fall Migrating—Georgia	2008	23
Fall Migrating—Georgia	2014	18
Overwintering—Mexico	2008	50
Overwintering—Mexico	2009	25
Overwintering—Mexico	2013	160
Grand Total		392

**Fig 3:**
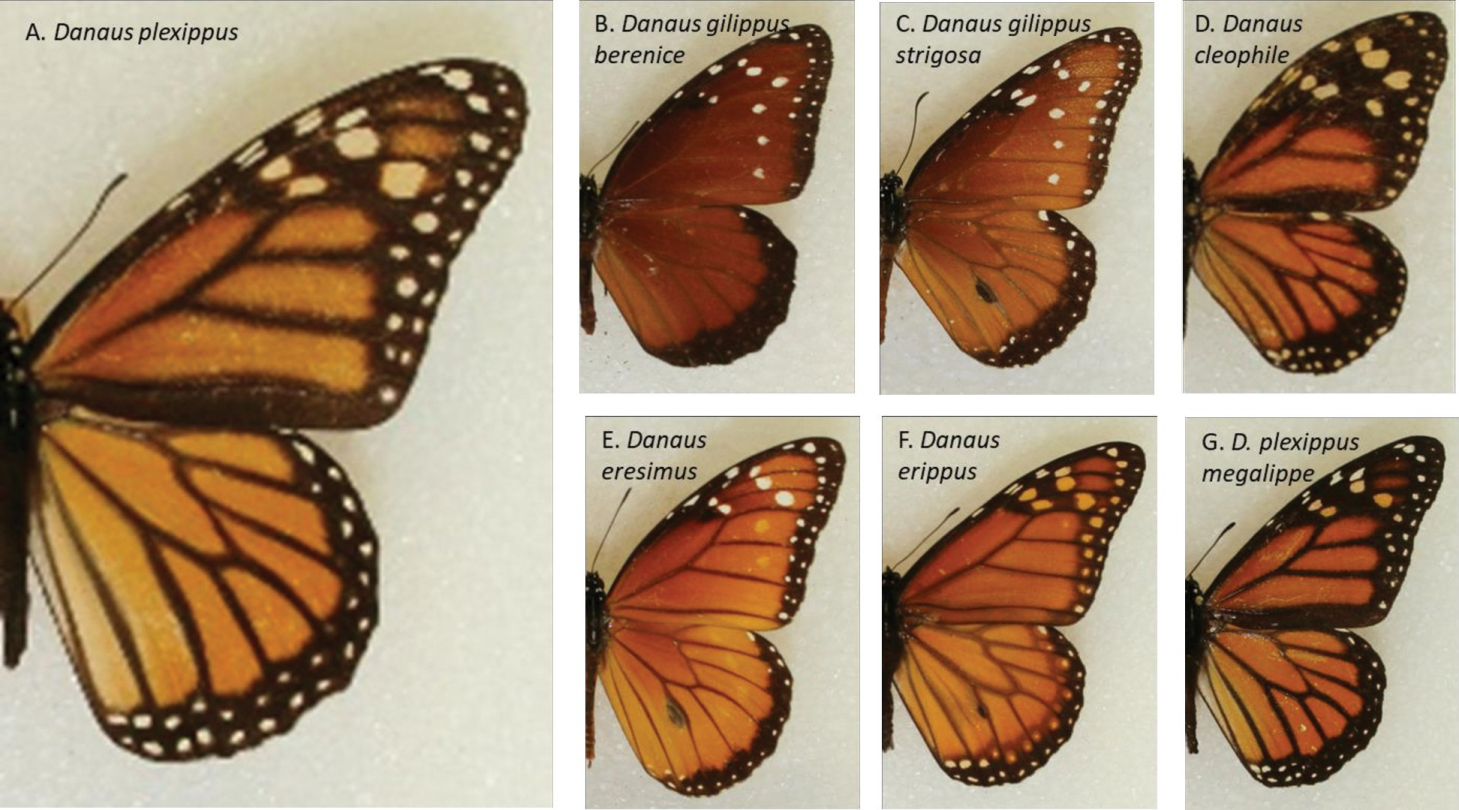
Comparison of wing patterns across seven closely-related Danaid butterflies examined in this study, including monarch (*Danaus plexippus*, A), Florida queen (*D*. *gilippus berenice*, B), striated queen (*D*. *gilippus strigosa*, C), Carribean queen (*D*. *cleophile*, D), soldier (*D*. *eresimus*, E), South American monarch (*D*. *erippus*, F), and *Danaus plexippus megalippe*, G. All images are photographs of pinned museum specimens, taken by P. Barriga.

As part of this project, we also examined images of 6 species or subspecies of Danaid butterflies that are close relatives to monarchs and that have ranges within the Americas, but are largely non-migratory. This was to ascertain if wing characteristics of North American monarchs (migratory) show any degree of divergence from that of their non-migratory relatives, which would be evidence of adaptation to the migratory lifestyle. These were photographs of pinned specimens from the American Museum of Natural History, which had been obtained for other purposes, by one of the authors (PB, and see S1 Fig in S1 File). These included *Danaus cleophile* (Jamaican monarch), *Danaus erippus* (southern monarch), *Danaus erisimus thetys* (soldier or tropical queen), *Danaus gilippus berenice* (queen), *Danaus gilippus strigosa* (striated queen), and *Danaus plexippus megalippe* (see Table 2 for sample sizes). These species all bear a superficial resemblance to monarchs, having similar base orange color, with black margins (Fig 3). These specimens were photographed at the museum using a digital camera mounted to a stand under uniform lighting (see S1 File for a photo of the procedure [S1 Fig] and details).

**Table 2:** Summary of pinned Danaid butterfly specimens examined from the American Museum of Natural History. All specimens were photographed at the museum by one of the authors (Barriga) as part of separate project. See Fig 3 for example images, and S1 Fig in S1 File for photography setup.

Species	Specimen origins	# Specimens
*Danaus cleophile* (Jamaican monarch)	Dominican Republic, Haiti, Jamaica	20
*Danaus erippus* (southern monarch)	Argentina, Brazil, Paraguay	46
*Danaus erisimus thetys* (soldier or tropical queen)	Cuba, Jamaica, Haiti, Dominican Republic	23
*Danaus gilippus berenice* (queen)	coastal Georgia and Florida (USA), Cuba, Bahamas	15
*Danaus gilippus strigosa* (striated queen)	coastal Georgia, Alabama and Texas (USA)	14
*Danaus plexippus magalippe*	Guatemala, Panama, Honduras	15
Grand Total		133

### Color analysis of monarchs

Our initial question was to ask if the amount (i.e. percentage) of any of the three basic wing colors—orange, black or white—are associated with migratory success in monarchs. To do this, we compared the relative surface area of each color among the three stages (breeding, migrating, and wintering) using the accumulated scanned monarch images (Table 1). We reasoned that if any one color was important for migration, then those monarchs that were successful in reaching the wintering colonies may have more (or less) of it than monarchs that had not undertaken the journey (breeding), or even those that were currently migrating. Measurements of color were done using an online color extraction tool called “Image Color Summarizer” (http://mkweb.bcgsc.ca/colorsummarizer/), which allows the user to input an image, and the program quantifies the amount of each color in the image based on a desired number of color samples and threshold of color difference. The color clusters are calculated using k-means clustering. These parameters were adjusted so that for each wing, the program returned the percentages of the three main colors (black, orange, and white) that were present on the wing. Note that a detailed description of this program, plus our specific menu selections, is provided in the S1 File associated with this paper.

### Measuring spot size

The results from the above analysis pointed to the importance of greater white pigmentation for migration success (see Results section), which led us to focus next on understanding the importance of the *marginal band of white spots* on monarchs and their relatives (see Fig 3). Specifically, we wanted to know if the size of these spots is associated with migration success, both within monarchs, as well as among migratory and non-migratory species (or subspecies). Here we measured both the scanned monarch images, plus the photographs of pinned Danaid specimens, in the same manner, and using the same image analysis procedure, described below. The fact that some images were of scanned wings and some were of photographed wings should not matter here, since the approach we used involved hand-tracing the spots, and then comparing the areas to the area of the wings. Thus, we used Adobe Photoshop (version 2021) with the Quantitative Image Analysis (QIA-64, www.reindeergraphics.com) plugins installed [52]. For each image we used the program’s magic wand tool to trace the outline of the right forewing, and then calculated the area, in pixels, of the wing using QIA menu selections. Next, we traced the outlines of all marginal spots on the same forewing (see. S2 Fig in S1 File), regardless of their color (though most were white), and calculated the collective area, in pixels, of these spots. From these two numbers, we obtained the relative size of all marginal spots (% of wing area) for each specimen, to use in analyses, below. This was repeated for all scanned monarch specimens, and for all photographs of Danaid specimens from the museum.

### Data analysis

For the first of the investigations outlined here, our goal was to determine if there is any variation or change in the amount of surface area covered by the monarch’s three primary wing colors (orange, black or white), across the breeding, migrating or wintering phase. Here, the percentage of orange, percentage of black, and percentage of white were response variables. These variables were normally-distributed, based on visual inspection of histograms. Since these proportions were inherently related, we used a MANOVA model to examine the variation in these parameters simultaneously, where stage was a categorical predictor (breeding, migration, or wintering), as well as the sex of the specimen. Since there were multiple collection years in each stage, we included year as a random effect. Following this test, we used univariate ANOVA models to separately ask how these predictors affected each color percentage by itself (response).

The second goal was to understand how marginal wing spot size (% of forewing area) varies among different species or subspecies of Danaid butterflies. The spot size data were normally-distributed, based on visual inspection of a histogram. We examined variation within these data using an ANOVA model, where the relative marginal spot size was the response, and species was the predictor. Included in this test were data from the 6 different Danaid species or subspecies (i.e. photographs of museum specimens, n = 133), plus from the collection of scanned monarch specimens (n = 392), which we separated into their three life stages (breeding, migrating, and wintering), for a total of 9 groups. This test essentially asked if the relative size of marginal spots of monarch butterflies differs from any of their close relatives, most of which are non-migratory.

All analyses were conducted using the Statistical 13.1 software package (Tibco Software, Inc).

## Results

**Fig 4:**
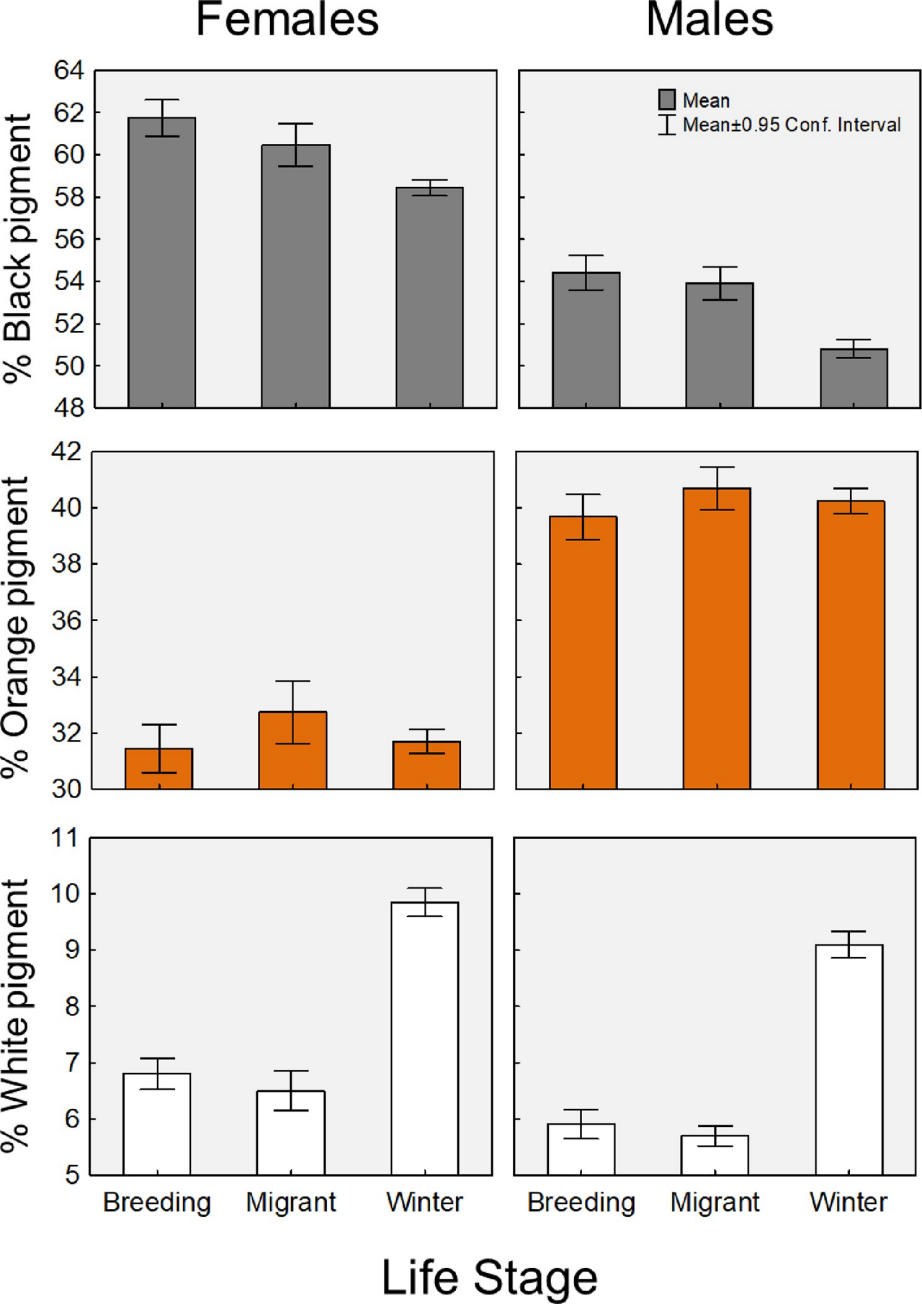
Plots of the extent of black, orange or white pigmentation on monarch specimens collected during the summer breeding season, the fall migration season, or from the overwintering sites in Mexico. Specimens were scanned and image analysis software used to measure the percent of each color on the right forewing.

The initial analysis of the 376 scanned monarch wings indicated that the relative surface area of two of the three primary colors varied across the summer, fall, and wintering stages (MANOVA: Wilks’ lambda, Λ = 0.640, approximate F_3,365_ = 68.4, P<0.0001; univariate tests: black: F_1,367_ = 35.8, P<0.0001; orange: F_1,367_ = 1.76, P = 0.186; white: F_1,367_ = 188.1, P<0.0001). A full model summary is presented in the S1 Table in S1 File, including results of univariate ANOVA models that examined effects on each color independently. According to these model results, the average percentage of the monarch forewing that was covered by black pigment declined by approximately 3% from the summer breeding stage to the overwintering stage (Fig 4). Meanwhile, the percentage of orange on the wings did not vary across migration stages. Finally, the average percentage of white on the forewing increased by approximately 3% from the summer to the wintering period. In sum, these comparisons revealed that lesser percentages of black, and greater percentages of white pigment on wings are associated with migration success in monarchs.

**Fig 5:**
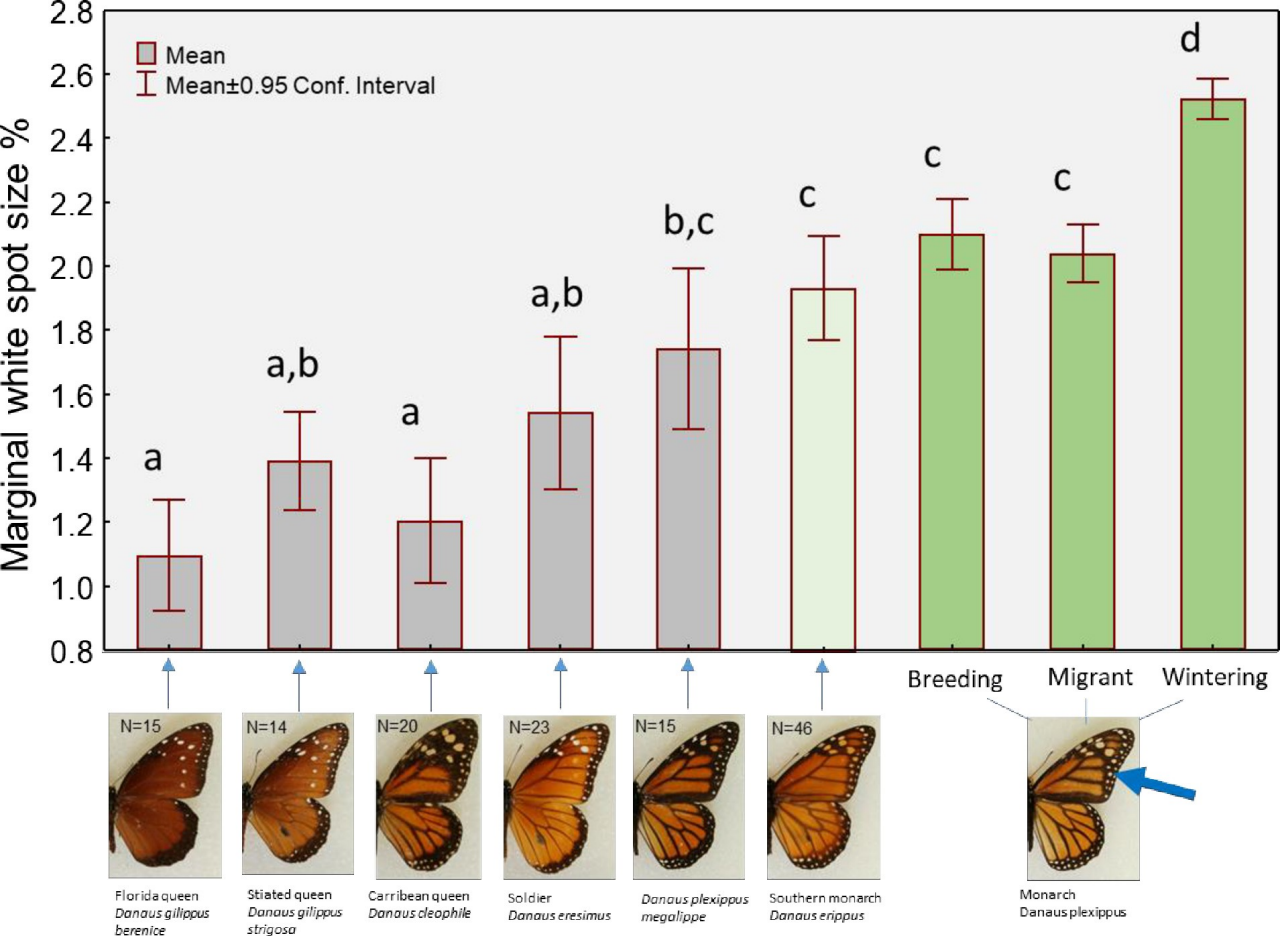
Comparison of relative white spot size on butterfly forewings (% of forewing surface area), for all Danaid species examined. There was significant variation in the means of groups in a one-way ANOVA (see results). Letters above bars indicate statistically significant groupings, based on Tukey’s post-hoc tests. North American monarch (*D*. *plexippus*) and southern monarch (*D*. *erippus*) are migratory and shown in green.

Comparison of marginal wing spot sizes among 6 Danaid species (and subspecies) and monarchs revealed significant variation in means across species (ANOVA, F_8,516_ = 50.45, p<0.0001), which is displayed in Fig 5. Tukey’s post-hoc tests revealed where group means differed significantly (denoted in figure); monarchs generally had the largest spots, followed by the semi-migratory *D*. *erippus*, which also had larger spots than all other (non-migratory) Danaids. Note that within the monarch groups, those that were collected at the wintering colonies had significantly larger marginal spots than those of summer breeding monarchs or even migrating monarchs (which had been collected in Georgia), which is consistent with the prior findings based on color alone. Collectively, these data argue that larger marginal spot size is an adaptation for migration.

## Discussion

In this investigation, we set out to elucidate if the primary wing colors of monarch butterflies in North America are associated with their famed long-distance annual migration. Given that recent studies with other animals, plus preliminary work on monarchs, all showed that black wing surfaces can provide aerodynamic benefits [34, 35, 37], we sought to determine first if the degree (or amount) of black pigment on monarch wings was associated with migration success. This turned out to be the case, although not in the direction we anticipated; our comparisons of colors from monarchs at three different stages of the flyway revealed that successful migrants tend to have slightly *less* black on their wings (by about 3%) than those who have not undertaken or finished the journey. This is the opposite of that found in a prior study focused on the monarch spring migration, where those individuals that had traveled the farthest tended to have *more* black coverage [29]. The inconsistency between these studies is notable, and it may stem from the environmental differences experienced by monarchs during the spring recolonization phase (especially solar exposure) compared to the fall. We also point out that the prior study compared melanism levels of monarchs that were raised in different regions of the flyway, and this may have confounded “migration distance” with varying rearing conditions/environments. Monarchs are known to develop darker wings when reared in cool environments [45]. Or, both conclusions could be sound, which means that monarchs really do experience dueling selective pressures on their wing melanism–the fall migration selects for reduced wing blackness in the southward-travelling cohort, and then the next generation (traveling northward in the spring) experiences selection for greater amounts of black.

Our primary wing color analysis also revealed that butterflies which succeeded in reaching Mexico also tended to have greater amounts of white pigment (i.e. marginal wing spots), by the same magnitude as the reduction in their black. Further, our examination of museum specimens revealed that migratory monarchs from North America have larger relative white wing spots than all other closely-related species or subspecies, most which do not migrate. The only other (partially) migratory species (*Danaus erippus*) in fact had the next largest spots, relative to its wing area. While we acknowledge that our pool of species (and subspecies) is small (n = 7), this evidence does argue that larger white spots are associated with the evolution of migration behavior within this group of New World butterflies. Combined, these findings from two separate, but related investigations, strongly suggests that the monarchs’ long-distance migration itself seems to select for larger marginal white spots every fall, so that only those individuals with large white spots will successfully reach their wintering destination, and thereby survive to pass on their genes to the next generation. This would lead to maintenance of large spots in migratory populations.

To be fair, while our collective results make a strong argument, it is possible that the spot size differences we observed across species are not specifically due to their migratory or non-migratory lifestyles, but that the light-colored spots simply act to counter-balance heat buildup from the melanic wing surfaces of all Danaids, even in sedentary species. Consider that the non-migratory Jamaican monarch, *D*. *cleophile*, has the most (relative) black pigment of all species (Fig 3), but it also has large inner white spots within that region (though its outer spots are small, Fig 5). This implies a need to counter-balance black pigment with white even in non-migratory species. In addition, our comparisons of monarch scans revealed that female monarchs, which naturally have more black wing pigment than males, also have significantly larger relative white spots than males (Fig 4, bottom panel). We estimate this difference to be approximately 23%, based on means of each sex across all time periods. To our knowledge, this basic morphological sex difference has not been described before in this otherwise well-studied butterfly species, though this may relate to the fact that we used fine-scale image analysis tools to examine these spots. In any case, this sex difference within monarchs also supports the idea that the white pigment is simply needed to compensate for the solar heat gain from the black pigment, and the more black pigment present on wings, the more white that is needed.

The fact that white pigment is known to serve a cooling function in wings of other butterfly species [41] led us initially to believe that the marginal spots of monarchs could also serve a similar function during the migration, when monarchs are soaring and exposed to direct solar energy. Our thinking was that this would then give those monarchs with larger white spots an advantage during the fall migration, since those with small spots may risk overheating. However, several pieces of evidence are inconsistent with this idea. First, the spots are not positioned anywhere near the butterfly body, the place where actual overheating is a factor [53]. Second, close examination of the position of the spots on the wings indicates that none actually overlap any wing veins, where they would be able to cool the flowing hemolymph [54]. Finally, we had even attempted to measure the thermal impact of white spots directly in our lab, but results of that effort (not included here) were not conclusive. If anything, our measurements showed how the spots provide little cooling, and only to the wing margin itself (Herkenhoff, unpubl data). Therefore, we dismiss the idea that the white spots serve to keep monarchs cool during migration.

Our working hypothesis is that the white spots have a role in the physical mechanics of flight aerodynamics, which is a research focus of two of the authors (Hassanalian, Herkenhoff). This hypothesis is based on recent experimental work from that lab that has investigated effects of pigmentation on flight of other flying animals (birds). Specifically, wind-tunnel studies of fixed wings with embedded heating elements revealed that heating the air immediately above the trailing edge of a wing created micro-vortices of air which reduced drag by as much as 20% [33]. In fact, this exact position (trailing edge) provided the most efficient means of improving flight performance compared to other heating locations on a model wing. This fact is noteworthy since monarch wings have a prominent black margin which forms the “trailing edge” when the butterflies are in a V-shaped soaring position (see Fig 1B). In theory, this black margin would provide a localized zone of heated (rising) air because of how black color absorbs solar radiation, and this could act to reduce drag in the same manner. In addition, the same study of birds also revealed how alternating black-and-white color patterns on wings can improve aerodynamics [33]. The cause of this improvement was not investigated specifically, but it is noteworthy that the majority of white spots in monarch wings are positioned along the black margin of the fore- and hindwings, thereby creating micro-zones of alternating black and white pigment. Possibly, monarchs may be making use of both the trailing edge heating effect, plus the black and white effect, to bolster aerodynamic performance. We have plans to further investigate these ideas using model butterfly wings in wind tunnels.

If the monarch’s white wing spots do play a role in aerodynamic function, and therefore improve migration success, this would not be altogether surprising. In fact, the idea that the North American monarchs’ long-distance migration could impose a selective force on wing spot size would not be dissimilar to how the migration maintains large wing sizes in this migratory population [25, 27, 28, 55]. Since large-winged monarchs are thought to have an advantage from being able to soar more effortlessly (and vice versa), small-winged monarchs are less likely to survive the long journey, and thereby reproduce the following spring. This explains why non-migratory populations of monarchs around the globe tend to have small wings, because the annual selective force of migration is gone [26, 56]. Similarly, there is also evidence that forewing shape (pointedness) is important for migration success in monarchs [7, 55], because of its own positive impact on flight performance, and this too is thought to undergo natural selection during the migration. It follows then, that *any* wing traits that convey an aerodynamic advantage (such as white spot size), should be favored during the long-distance migration, and ultimately lead to morphological differences between migrant and resident species.

Given the complete lack of information on the role of the white marginal spots on monarchs and other Danaid butterflies, it is important to consider other possible explanations for the patterns we observed, both within the monarch scans (Fig 4) and the comparisons across species and subspecies (Fig 5). One explanation in particular is that spots may have a role in predator-defense. Specifically, certain bird species are known to predate on overwintering monarchs [57], and, if the larger white spots offer any protection from this, it may explain why spots tend to be larger in this stage. This may also explain why monarchs, which face this annual threat, have evolved larger spots than other Danaids, which do not ever form large, susceptible overwintering clusters. Interestingly, there is at least some evidence that white-colored wings act as signals of unpalatability in field experiments with *Pieris* and *Colias* butterflies [58]. Future efforts should be made to ascertain if white wing spots of monarchs also convey unpalatability. Thus far, only the aposematic orange and black colors of monarchs has been thought to convey their distastefulness [59].

**Fig 6:**
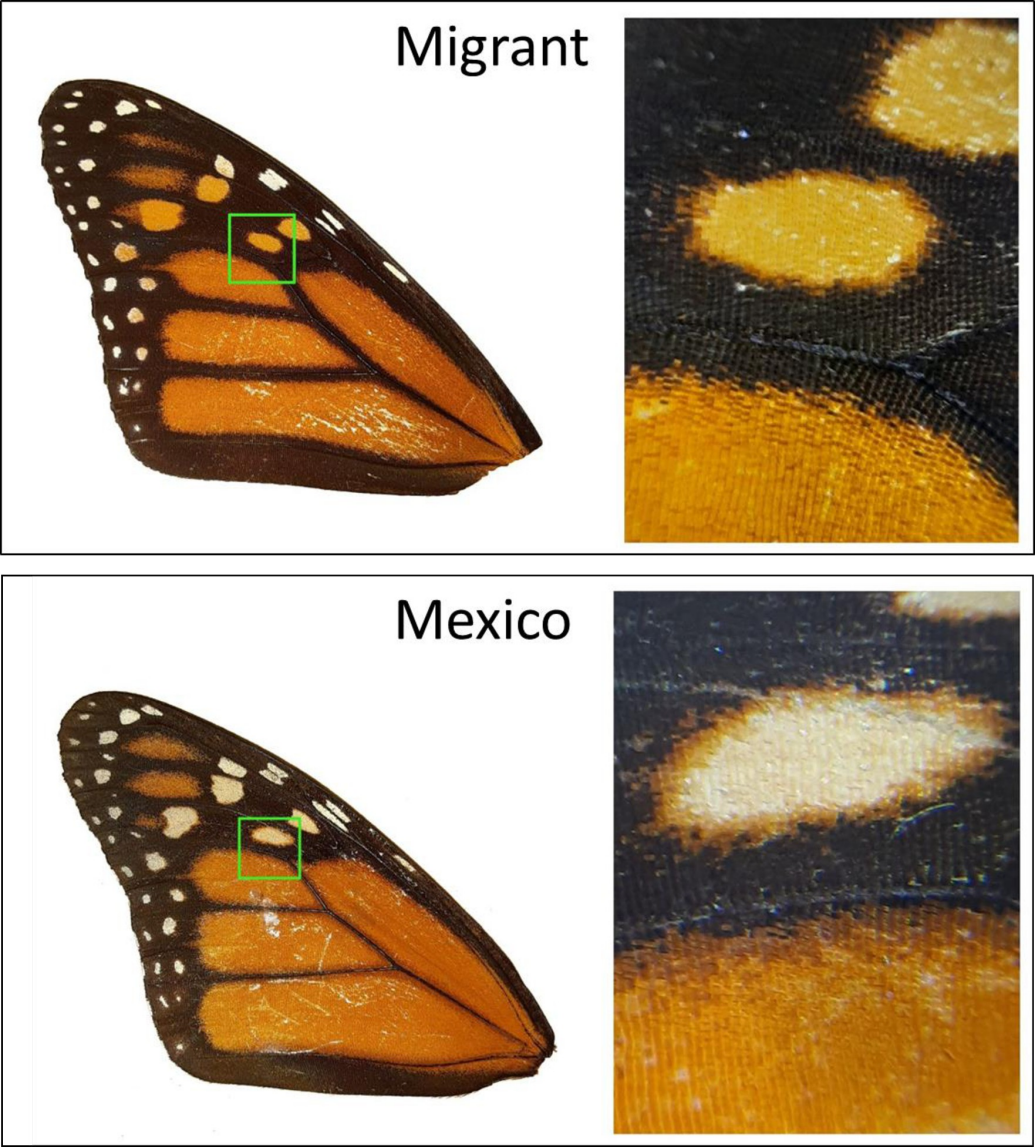
Scanned forewings of monarchs collected at different stages of the migratory journey, for close-up comparison of spot colors. Top image is the forewing of a monarch collected in Georgia during the fall migration, with one central spot magnified (right). Below is a forewing of a monarch that successfully reached the overwintering colony, with the same spot magnified (right).

Yet another alternative explanation for the greater amount of white found in wintering monarchs, is that perhaps during the long flight their wing spots become bleached from the sun, thus creating the “greater” amount of white we observed. Indeed, bleaching of wing colors is thought to occur during the monarch migration [60]. At this time, we have no data to discount this possibility, though close examination of wings from breeding vs. wintering monarchs (Fig 6) does in fact discount this idea. It is apparent when closely looking at the same spots in monarchs from both stages that the wintering monarch have “white” spots with a white center, but also with a distinct orange edge. If this spot became white from sun-bleaching, it would have had to only bleach in the center, which seems unlikely. Bleaching should result in the fading of all pigments. We therefore are inclined to dismiss this idea until further research can be done to evaluate how much the wing pigments can change over time, perhaps with experimentation. In addition, this “bleaching” explanation would not explain why monarch spots are physically larger than their close relatives (Fig 5).

Clearly, this research begs many new questions about this “hidden function” of the charismatic wing color patterns of monarchs, and perhaps it will serve as a useful starting point for additional research, both in the biological, as well as aerospace engineering fields. While the idea that black pigmentation (melanism) can enhance warm-up speeds of butterflies in cool environments has been around for a while [61, 62], the notion that non-melanic spot patterns would be also important for long-distance flight is, to our knowledge, novel.

## Conclusions

The perilous long-distance migration of North American monarchs exerts an annual selective force, allowing only those individuals with traits that promote long-distance flight to survive. We provide evidence here that the marginal white wing spots of monarchs appear to be important for successful migration. First, we show that those individuals that succeed in reaching the wintering colonies in the fall have significantly more white pigment, plus larger marginal spots, relative to their wing size, than those at other stages of the journey. Further, the North American migratory monarch population (and the partially migratory southern monarch) appears to have the largest marginal spots compared to related, New World, Danaid butterflies, suggesting that large wing spots are a trait that has evolved with migration behavior. Other explanations for this pattern are possible, and future work is needed. Collectively, these findings offer fresh perspectives on not only how monarchs are able to successfully fly thousands of kilometers annually, but also on how monarchs “got their spots.”

## Supporting information

S1 File Supplemental file containing additional figures, description of procedures and model results. In order, the supplemental file contains: S1 Fig, description of procedure for measuring colors of monarch wings, S2 Fig, and S1 Table.
(DOCX)

S1 Data Davis et al raw data. Complete excel dataset of all raw data used in analyses.
(XLSX)
