# Supplementary material for: How the monarch got its spots: Long-distance migration selects for larger white spots on monarch butterfly wings
Source: PLoS One. 2023 Jun 21;18(6):e0286921. doi: 10.1371/journal.pone.0286921 (PMC10284392; doi:10.1371/journal.pone.0286921)
Supplement: S1 File — In order, the supplemental file contains: S1 Fig, description of procedure for measuring colors of monarch wings, S2 Fig, and S1 Table. (DOCX) [file pone.0286921.s001.docx]

**How the monarch got its spots: long-distance migration selects for larger white spots on monarch butterfly wings**

**Supplemental file**

This document contains:

- Fig. S1 – Photo of author, Barriga, while photographing butterfly specimens in the American Museum of Natural History in New York, NY USA
- Description of quantification of primary wing colors of monarch butterflies across three life stages (breeding, migration, overwintering).
- Fig. S2 – Description of marginal spot measurement on butterfly images.
- Table S1 - Summary of MANOVA model, and follow-up ANOVA models, that examined predictors of color variation in monarchs across their summer, fall or winter periods

Figure S1. (Left) photo of author, Barriga, while photographing butterfly specimens in the American Museum of Natural History in New York, NY USA. The photography room was apart from the museum and had standard florescent lighting. Right photo shows the camera setup for photographing butterfly specimens. The camera was a Cannon Rebel XT with EFS 18-55mm lens, and it was mounted to a copy-stand with an adjustable height. No flash was used to illuminate the specimens, so as to replicate their natural color. All pinned butterfly specimens were placed in a specimen box with a white background, and with a ruler visible in the image.


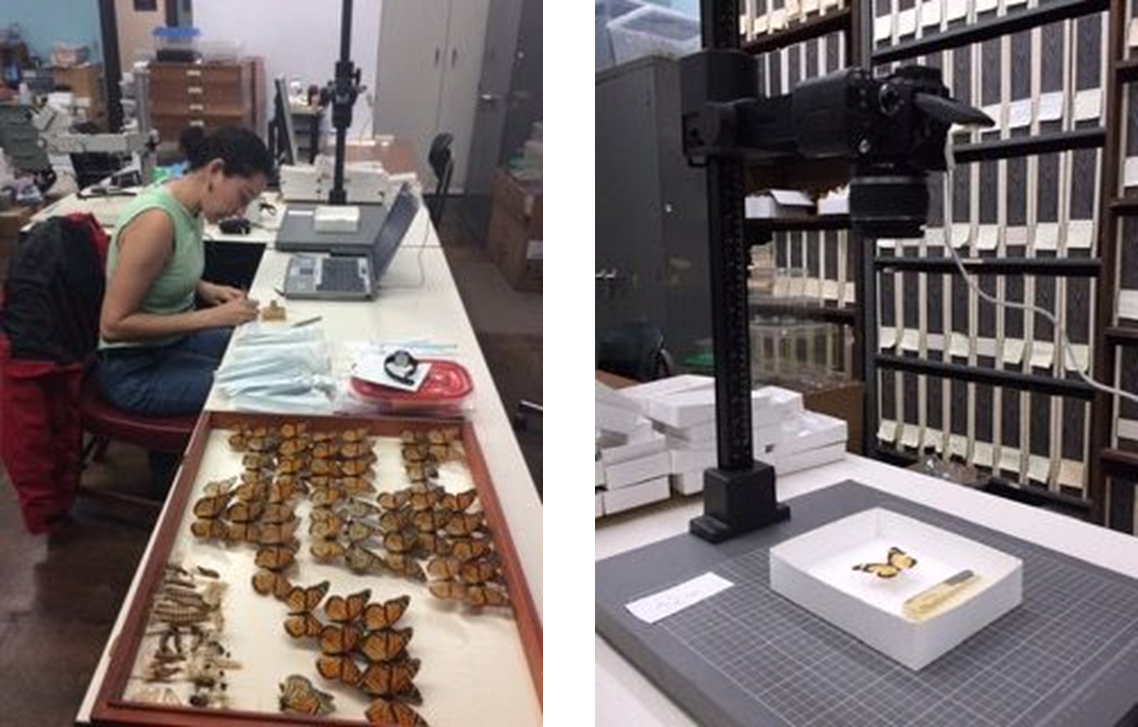


**Description of quantification of primary wing colors of monarch butterflies across life stages**

For the first phase of this project, we were interested in understanding how the primary wing colors of monarchs (orange, black or white) varied across three life stages - breeding, migration, or overwintering, and we quantified colors on 375 scanned forewings. For each wing scan we initially swapped the background with green, so as to be able to isolate the primary colors.

We used a free online website tool for these color analyses, called “Image Color Summarizer” - <http://mkweb.bcgsc.ca/colorsummarizer/>, which allows the user to input an image, and the program quantifies the amount of each color. The colors can be selected, and the specificity of the color-selector can be adjusted. Below are screenshots with the values we used indicated. The color clusters are calculated using k-means clustering (<https://en.wikipedia.org/wiki/K-means_clustering>).

1. Select Analysis tool


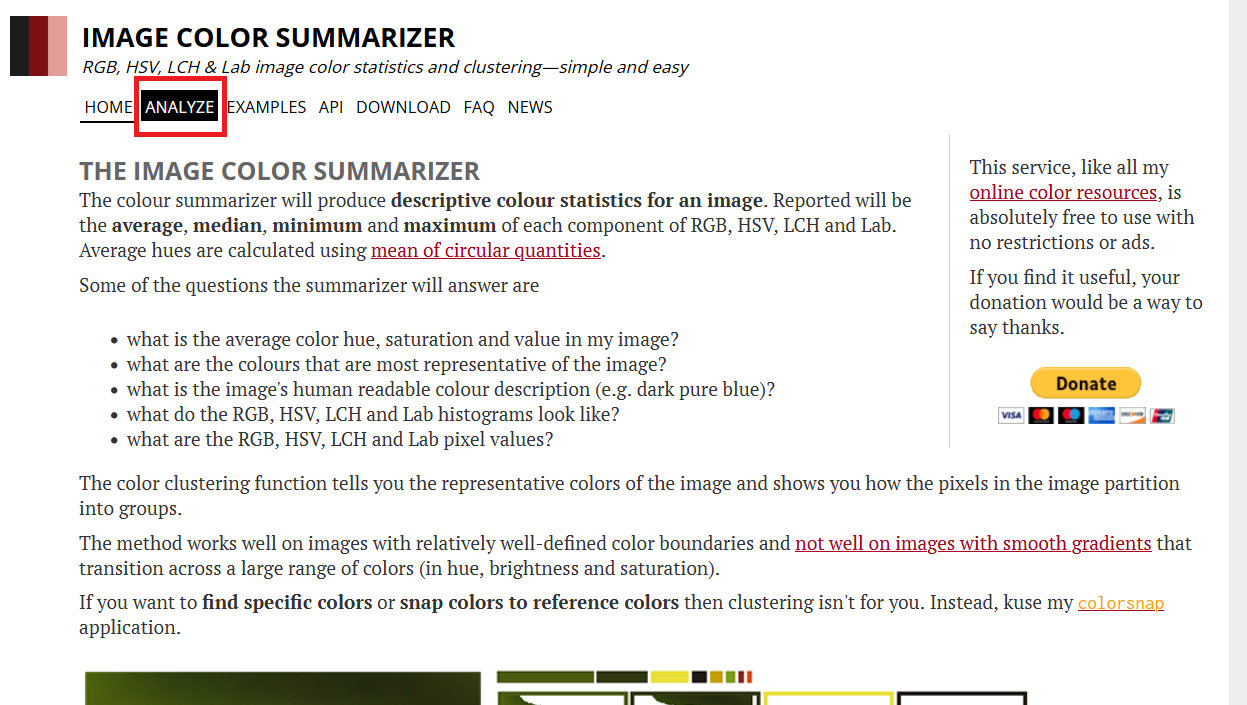


1. Insert forewing images with green background (a green background allowed us to isolate the background color from the white colors on the wing). Then we specified we wished to quantify the amount of 4 colors (Black, Orange, Grey, and Green, see screenshot image).


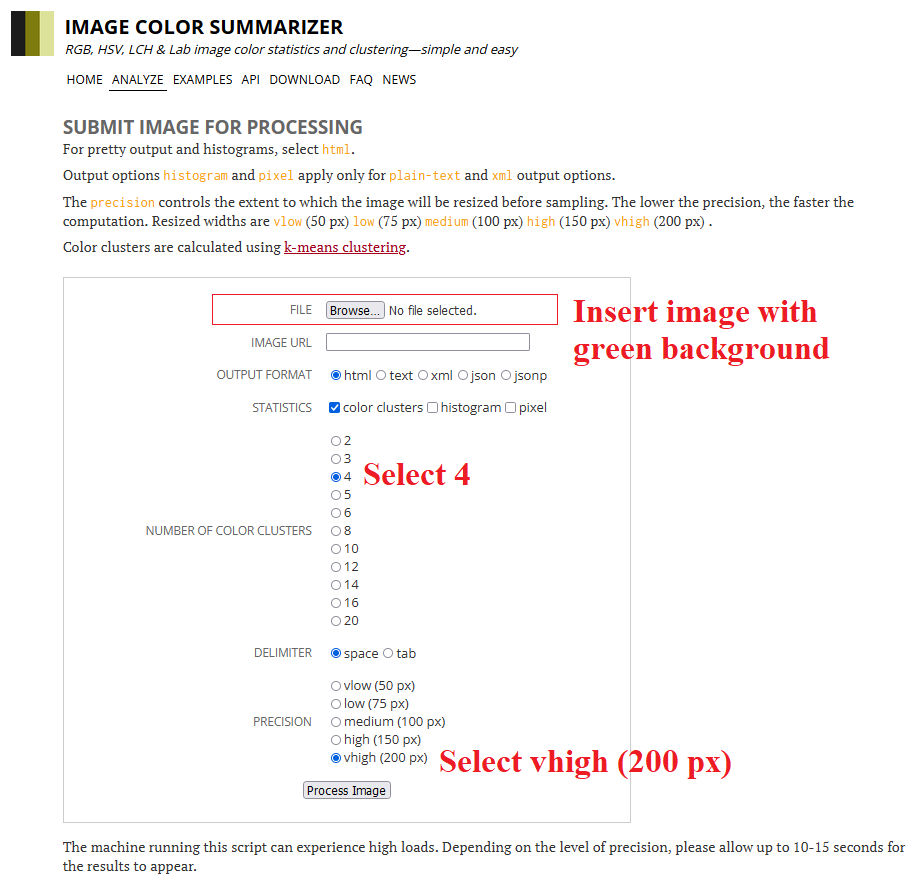


1. Press process Image


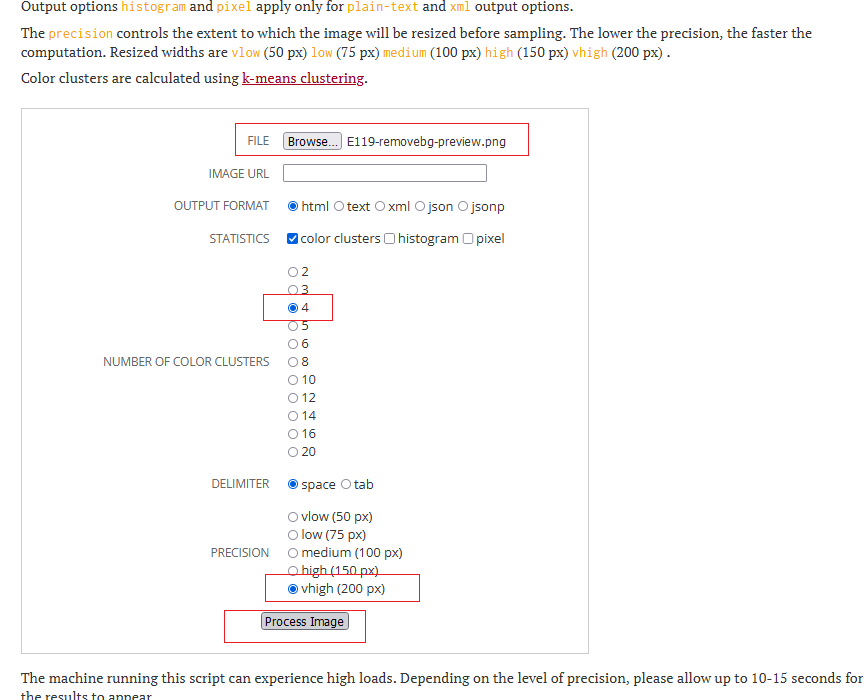


1. Results are displayed, indicated by the highlighted square. The program displays the percentage of the image that contains the colors specified. We then removed the green (background).


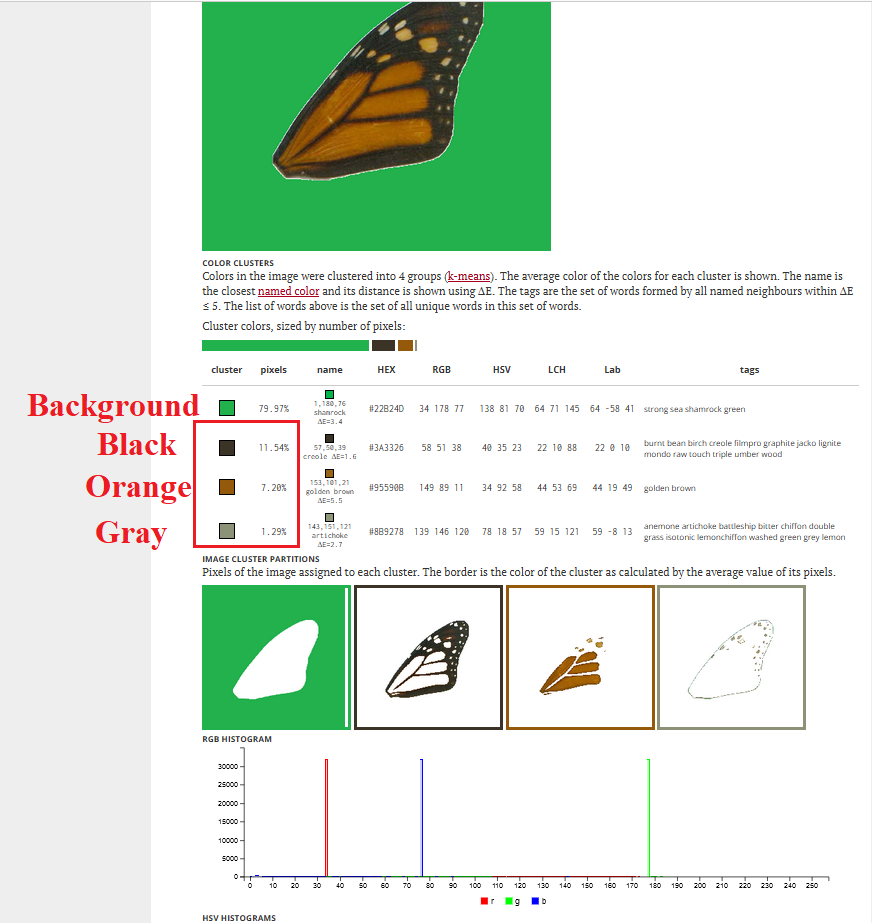


Figure S2. Description of marginal spot **area** measurement on scanned butterfly wings. Forewing images of monarchs and related Danaid species were imported into Adobe Photoshop, with the Quantitative Image Analysis software plugins installed ([www.reindeergraphics.com](http://www.reindeergraphics.com)). The user used the magic want tool to select each marginal spot, regardless of its color (right panel). A menu routine was used to calculate the area of the spots (total number of pixels). This, plus the area of the wing (also in pixels) was used to calculate the relative surface area (%) taken up by all marginal spots.


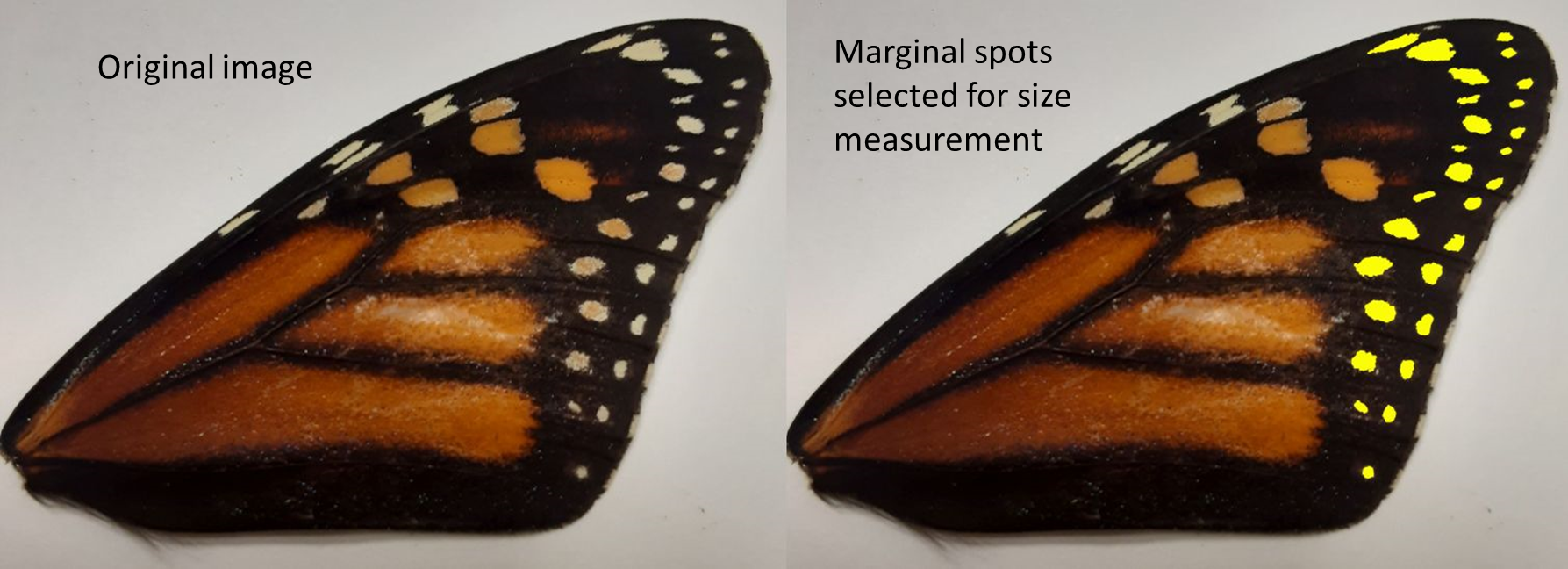


Table S1. Summary of MANOVA model that examined predictors of color variation in monarchs across their summer, fall or winter periods. A total of 392 monarch wings were measured. In the full model (initial table), the percentage of each color (black, orange and white) were included as response variables. The second table shows results of models where each color was the sole response variable in an ANOVA model with the same predictors.

Full model summary

| Predictor | Wilks lambda | F | Effect df | Error df | p |
| --- | --- | --- | --- | --- | --- |
| Sex | 0.232 | 403.8 | 3 | 365.0 | <0.0001 |
| Stage | 0.640 | 68.4 | 3 | 365.0 | <0.0001 |
| Year | 0.815 | 5.2 | 15 | 1008.0 | <0.0001 |

Univariate results

|  |  |  | | % black pigment | | |  | |  | | % orange pigment | |  | | |  | | % white pigment | |  | | |  |
| --- | --- | --- | --- | --- | --- | --- | --- | --- | --- | --- | --- | --- | --- | --- | --- | --- | --- | --- | --- | --- | --- | --- | --- |
| Predictor | Effect | df | SS | | MS | F | | p | | SS | | MS | | F | p | | SS | | MS | | F | p | |
| Sex | Fixed | 1 | 5032.9 | | 5032.9 | 1004.3 | | 0.0000 | | 6453.1 | | 6453.1 | | 1169.00 | 0.0000 | | 55.78 | | 55.78 | | 47.95 | 0.0000 | |
| Stage | Fixed | 1 | 179.6 | | 179.6 | 35.8 | | 0.0000 | | 9.7 | | 9.7 | | 1.76 | 0.1859 | | 218.81 | | 218.81 | | 188.10 | 0.0000 | |
| Year | Random | 5 | 228.7 | | 45.7 | 9.1 | | 0.0000 | | 275.0 | | 55.0 | | 9.96 | 0.0000 | | 27.14 | | 5.43 | | 4.67 | 0.0004 | |
| Error |  | 367 | 1839.1 | | 5.0 |  | |  | | 2025.9 | | 5.5 | |  |  | | 426.92 | | 1.16 | |  |  | |
| Total |  |  | 7714.4 | |  |  | |  | | 8944.1 | |  | |  |  | | 1507.83 | |  | |  |  | |
